# DNA methylation profiles in ovarian cancer: Implication in diagnosis and therapy (Review)

**DOI:** 10.3892/mmr.2014.2221

**Published:** 2014-05-08

**Authors:** OURANIA KOUKOURA, DEMETRIOS A. SPANDIDOS, ALEXANDROS DAPONTE, STAVROS SIFAKIS

**Affiliations:** 1 Department of Obstetrics and Gynecology, University Hospital of Larissa, Larissa, Thessaly, Greece; 2 Laboratory of Clinical Virology, University of Crete Medical School, Heraklion, Crete, Greece; 3 Department of Obstetrics and Gynecology, University Hospital of Heraklion, Heraklion, Crete, Greece

**Keywords:** biomarkers, DNA methylation, epigenetic mechanisms, oncogenes, ovarian cancer, tumor suppressor genes

## Abstract

Genetic alterations alone cannot account for the complexity of ovarian cancer. The potential reversibility of epigenetic mechanisms makes them attractive candidates for the prevention and/or treatment of ovarian carcinoma. Detection of the epigenetic signature of each cancer cell may be useful in the identification of candidate biomarkers for disease detection, classification and monitoring and may also facilitate personalized cancer treatment. In ovarian cancer, in addition to other non-gynaecological cancers, two opposite epigenetic phenomena occur. The first involves an overall global decrease in DNA methylation of heterochromatin leading to demethylation of several oncogenes, while the second involves specific CpG island hypermethylation associated with the promoters of tumor suppressor genes. Early studies focused on the methylation patterns of single genes associated with tumorigenesis. However, newer genome-wide methods have identified a group of genes whose regulation is altered by DNA methylation during ovarian cancer progression.

## 1. Introduction

Ovarian cancer is the leading cause of gynecologic cancer death, while constituting only 3% of all female cancers (1). Although the exact cause of ovarian malignancies remains unknown, the fact that >50% of deaths occur in postmenopausal women aged 55–74 years, suggests a hormonal risk. Due to the lack of specific symptoms in early stage, 70% of cases are not diagnosed until the cancer has reached an advanced stage, FIGO Stages IIB to IV (spread of tumor within the pelvis or elsewhere in the abdomen) (2). Early detection of ovarian cancer reportedly increases the five-year survival rate by up to 92%; however, the actual overall five-year survival rate is only 15–45% (3). Despite advances in cancer research and treatment, these survival statistics have remained largely unchanged for many years. The lack of early detection markers and the development of drug resistance following chemotherapy, are the main obstacles to effective treatment strategies. A better understanding of the molecular pathogenesis of ovarian cancer is needed in order to develop new drug therapies or diagnostic biomarkers and elucidate the role of environmental exposures to the individual’s predisposition to the disease.

Ovarian epithelial carcinoma (OEC) is the most common ovarian malignancy, with substantial histopathological heterogeneity. According to the 2003 World Health Organization classification scheme, the most common histologic subtype is serous ovarian carcinoma (~60%), while other subtypes include endometrioid (10–20%), clear cell (10%), transitional (6%), mucinous (<5%), and undifferentiated (<1%) subtypes (4). The underlying genetic basis of ovarian cancer contributes to this heterogeneity. The majority of OECs (90%) are sporadic, with the remaining OECs being inherited. Inherited ovarian cancers account for 5–10% of all ovarian cancers and are characterized by the development of highly aggressive neoplasms at an earlier age of onset than their sporadic counterparts (4). Mutations of *BRCA1* and *BRCA2* tumor suppressor genes are responsible for most hereditary ovarian cancers. The two genes are essential for DNA repair and play integral roles in genomic stability and integrity (5).

A number of studies (6–8) have reported the use of the candidate gene approach in the search for common risk variants associated with ovarian cancer. Identification of common genetic susceptibility alleles may lead to a greater understanding of disease etiology, potentially leading to genetic screening approach that could be used to identify the proportion of the population that would benefit from screening. Genes have been selected from relevant biological pathways, steroid hormone metabolism, DNA repair, apoptosis and cell cycle control, as well as known oncogenes and tumor suppressor genes. However, the genes that participate in the development of ovarian cancer represent only a small portion of the ovarian cancer-associated genes, as many of them are merely associated with ovarian cancer development but do not contribute to its initiation and progression. Moreover, molecular pathways in different ovarian tumors may vary significantly. Thus, genetic alterations alone cannot account for the complexity of ovarian cancer. Since genetic factors are almost impossible to reverse, the potential reversibility of epigenetic mechanisms makes them attractive candidates for the prevention and/or treatment of ovarian carcinoma (9–11).

Epigenetic mechanisms are heritable changes in gene expression without altering the primary DNA sequence (12). Epigenetics involves the interplay between DNA methylation, histone modifications and expression of non-coding RNAs in the regulation of gene transcription (13). Increasing evidence has shown that epigenetic alterations including DNA methylation play a significant role in cancer, from the silencing of tumor suppressors to the activation of oncogenes and the promotion of metastasis (14). DNA methylation is a key element in tissue differentiation during early embryonic development. The diversion of a normal cell cycle to those of a less differentiated status comprises one of the initial steps of tumorigenesis (15). Aberrant DNA methylation is now recognized as one of the most common molecular abnormalities in cancer frequently associated with drug resistance (14).

DNA methylation comprises the best known epigenetic mechanism associated with gene expression. DNA methylation occurs on the cytosine residues of CG (also designated as CpG) dinucleotides. Enzymes known as DNA methyltransferases (DNMTs) catalyse the addition of a methyl group to the cytosine ring to form methyl cytosine, employing S-adenosylmethionine as a methyl donor (16). In humans and other mammals, DNA modification occurs predominantly on cytosines that precede a guanosine in the DNA sequence (16). These dinucleotides can be clustered in small stretches of DNA, termed CpG islands, which are often associated with promoter regions. Most CpG sites outside the CpG islands are methylated, suggesting a role in the global maintenance of the genome, while most CpG islands in gene promoters are unmethylated, which allows active gene transcription (16,17). Generally, when a given stretch of cytosines in a CpG island located in the promoter region of a gene is methylated, that gene is silenced by methylation, and such a CpG island would be termed ‘hypermethylated’. Conversely, when a given stretch of cytosines in a CpG island located in the promoter region of a gene is not methylated, that gene is not silenced by methylation, and the CpG island in this case would be ‘hypomethylated’ (18). Methylation of promoters inhibits their recognition by transcription factors and RNA polymerase, as methylated cytosines preferentially bind to a protein known as methyl cytosine binding protein, or MeCP. When a promoter region normally recognized by an activating transcription factor, is methylated, its transcription is inhibited (19).

The DNA methylation profile of a tumor cell is a reflection of its somatic lineage, environmental exposure and genetic predisposition. The DNA methylation profile is therefore distinct for each histological subtype, suggesting different tumorigenic mechanisms. The detection of the epigenetic signature of each cancer cell may be useful in the identification of candidate biomarkers for disease detection, classification and monitoring and facilitate personalized cancer treatment.

## 2. DNA methylation in ovarian cancer

**Figure 1:**
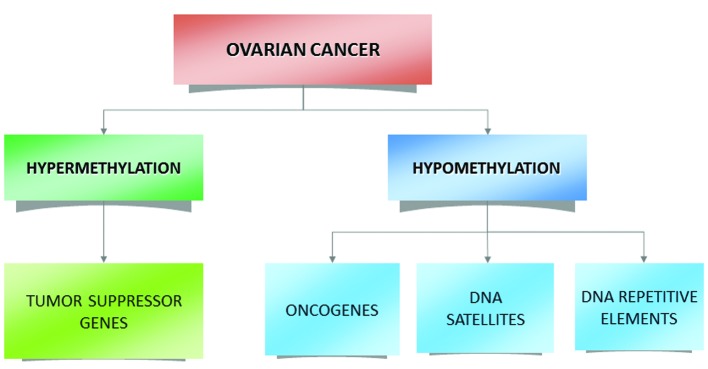
Schematic representation of the methylation events associated with ovarian tumorigenesis.

In ovarian cancer, in addition to other non-gynaecological cancers, two opposite epigenetic phenomena occur: i) An overall global decrease in DNA methylation of heterochromatin leading to demethylation of several oncogenes, ii) specific CpG island hypermethylation associated with the promoters of tumor suppressor genes (9,20–22) (Fig. 1). The aberrant methylation of CpG islands in gene promoters has been correlated with a loss of gene expression, and it appears that DNA methylation provides an alternative pathway to gene deletion or mutation for the loss of tumor suppressor gene (TSG) function (23). The epigenetic silencing of TSG induces such mechanisms as uncontrolled cell division, the ability to infiltrate surrounding tissues, metastasis, avoiding apoptosis or sustaining angiogenesis, all of which are responsible for promoting tumor development. In ovarian cancer, a large number of TSGs have been found to undergo hypermethylation (24–26).

One of the most studied genes in ovarian cancer is breast cancer early onset gene 1 (*BRCA1*) gene, due to its role in inherited and sporadic forms of the disease (27,28). *BRCA1* is important in maintaining genomic stability (29), and interacts with numerous proteins, forming complexes that are involved in recognizing and subsequently repairing DNA. Evidence suggests that in cases of sporadic ovarian cancer promoter hypermethylation, non-somatic mutation is the cause for *BRCA1* inactivation (30). Aberrant methylation of the gene promoter may also serve as an alternative explanation for the loss of heterozygosity associated with *BRCA1* deficiency in ovarian carcinomas (31). Complete or partial inactivation of the BRCA1 gene through hypermethylation of its promoter has been reported in 15% of sporadic ovarian tumors (27,32). Hypermethylation leads to the silencing of this gene in ovarian tumors and levels of methylation correlated with decreased *BRCA1* expression (33,34). Compared to stage I and healthy subjects, there were higher *BRCA1* promoter methylation frequencies in stage II and III ovarian cancers (34). In a series comparing the methylation status of *BRCA1* among tumor samples obtained from patients with benign ovarian tumors, borderline tumors as well as carcinomas, promoter methylation was detected in 31% of carcinomas but in none of the benign or borderline tumors (35). Hypermethylation of *BRCA1* was detected at a significantly higher frequency in serous carcinomas than in tumors of the other histological types (36). Of note, methylation of *BRCA1*, while frequent in sporadic ovarian cancer, it has not been reported in the hereditary type of the disease, nor in samples from women with a germ-line *BRCA1* mutation (37,38). *BRCA2* does not exhibit a similar methylation profile in ovarian cancer (39). Findings of previous studies have shown that methylated CpGs at the *BRCA2* promoter were either absent or at very low levels in tumor DNA compared to normal tissues (33).

A number of other classical TSGs have been found to undergo hypermethylation in cases of ovarian cancer. Tumor suppressor genes involved in DNA mismatch repair (MMR) have a distinct carcinogenic mechanism in ovarian tumors. DNA MMR is an endogenous molecular mechanism that reverses replication errors that escape correcting by replicative DNA polymerases. In MMR-defective cells, both base-to-base mismatches and insertion/deletion loops, are left uncorrected (40). This results in increased spontaneous somatic mutations. This effect is particularly obvious in non-expressed sequences comprising multiple simple repeats (microsatellites), and the characteristic microsatellite instability (MSI) is diagnostic for MMR-defective tumors (41,42). Approximately 10% of ovarian cancers are related to this molecular pathway (43). Defective MMR is often a consequence of germ-line mutations in the *hMLH1*, *hMSH2*, *MGMT* or, occasionally, *MSH6* or *PMS2* genes. Hypermethylation of the *MLH1* gene accompanied by loss of the gene expression has been reported in 10–30% of ovarian malignancies, while in cases with acquired resistance to platinum-based chemotherapy, *hMLH1* promoter methylation has been identified in 56% of cases (44,45). The methylation frequency of *hMSH2* promoters has been reported to be as high as 57% in ovarian cancers. Methylation of hMSH2 correlated with histological grade and lymphatic metastasis. Additionally, the methylation rates of hMSH2 were significantly higher in endometrioid adenocarcinoma tissues compared to other pathological types of the disease (44).

RAS association domain family protein 1a (RASSF1A) which is an inhibitor of the anaphase-promoting complex, together with *OPCML*, are among the most frequently methylated genes in ovarian cancer (46,47). Genes involved in cell cycle pathways such as p16 and p15 have also been affected by altered methylation of their promoters (48). E-cadherin is a transmembrane glycoprotein that mediates calcium-dependent interactions between adjacent epithelial cells. It has been found that the risk of E-cadherin hypermethylation was 1.347-fold among patients with ovarian cancer than that among patients with benign ovarian lesions (48). Other genes involved in cell adherence, such as H-cadherin and CDH1, have shown similar results (49). HSulf-1, which encodes an arylsulfatase that acts on cell surface heparin sulfate proteoglycans and inhibits growth factor signalling, was found to be methylated in >50% of ovarian tumors and cell lines (50).

Methylation profiles of several genes belonging in the family of the Homeobox (HOX) genes have also been investigated in cases of ovarian carcinomas. Homeobox genes constitute a family of transcription factors that function during embryonic development to control pattern formation, differentiation, and proliferation (51). *HOX* genes are expressed in normal adult reproductive tissue where they are involved in regulating differentiation. Findings of previous studies suggest that the abnormal expression of particular *HOX* genes is associated with ovarian cancers (52). Methylation of the *HOXA9* gene has been observed in 95% of patients with high grade serous ovarian carcinoma (53). It has been suggested that the methylation status of *HOXA9* and *HOXAD11* genes may serve as potential diagnostic and prognostic biomarkers (53,54).

The majority of studies assessing the methylation status of TSGs have focused on single genes with varying reported frequencies in different tissues. Hypermethylation in ovarian cancer, however, has been found to be associated with the inactivation of almost every pathway involved in ovarian cancer development, including DNA repair, cell cycle regulation, apoptosis, cell adherence and detoxification pathways (32,38,55–58).

In addition to the hypermethylation of promoter-associated CpG islands, global hypomethylation and specific hypomethylation of protein expressed genes that subsequently become overexpressed plays a significant role in ovarian cancer. Hypomethylation in the centromere and subtelomeric regions is involved in the induction of genomic instability (GI), leading to chromosomal translocations and gene disruption through the reactivation of transposable elements (21). Decreased methylation of LINE-1 elements is correlated with high grade, advanced stage and poor prognosis in ovarian cancer patients (59). Satellite DNA hypomethylation is an independent marker of poor prognosis. Hypomethylation is increased from non-neoplastic tissue toward ovarian cancer as well as advanced grade and stage (60).

In addition to repetitive elements and DNA satellites, a number of protein-coding genes are overexpressed in ovarian cancer, in association with promoter hypomethylation. Several oncogenes have been reported to have an increased epigenetically induced expression. Oncogenes such as *CLDN4* (encoding an integral component of tight junctions), *MAL* (mal, T-cell differentiation protein) and *BORIS* (brother of the regulator of imprinted sites) belong to a number of oncogenes that contribute to drug resistance and are associated with overall prognosis of the disease (61–63). Upregulation, together with hypomethylation of the ABCG2 multidrug transporter and *TUBB3* genes, which is a determinant of taxane resistance, have been observed in cases of advanced ovarian carcinoma with drug-acquired chemoresistance (64,65). Other cancer-associated genes including *MCJ* (66,67) and *SNGG* (synucelin-γ), encoding an activator of the MAPK and Elk-1 signaling cascades (63,68), are upregulated in ovarian cancer in association with DNA hypomethylation.

## 3. Diagnosis

Since aberrant methylation is one of the earliest molecular alterations during tumorigenesis, it has been suggested as a promising strategy for the early detection of ovarian cancer. However, methylation of single genes may have limited value in clinical applications. At present, no single epigenetic biomarker is able to accurately detect early ovarian cancer in either tissue or body fluids. Analysis of the methylation status of multiple genes simultaneously in a blood-based assay may provide a more sensitive and specific method for the molecular classification and prognosis of ovarian cancer.

A genome-wide DNAm profiling of a large ovarian cancer case control cohort demonstrated that active ovarian cancer has a significant impact on the DNAm pattern in peripheral blood (69). A microarray-based analysis on ovarian tumors identified 112 methylated loci prognostic for progression-free survival in advanced ovarian cancer patients (70). The data suggested that a higher degree of CpG island methylation is associated with early disease recurrence following chemotherapy (71). Promoter hypermethylation of at least one of six genes (*BRCA1*, *RASSF1A*, *APC*, *p14ARF*, *p16INK4A* and *DAPK*) was observed in 41/50 ovarian cancer serum specimens. Thus, hypermethylation of certain genes may present an early event in ovarian tumorigenesis that can be detected in the serum DNA from patients with ovary-confined (stage IA or B) tumors and in cytologically negative peritoneal fluid (56). A recent study that used multiplex methylation-specific PCR to analyze the methylation status of cell-free serum DNA of seven candidate genes (*APC*, *RASSF1A*, *CDH1*, *RUNX3*, *TFPI2*, *SFRP5* and *OPCML*), achieved a sensitivity and specificity of 85.3 and 90.5%, respectively, in stage I OEC. The detection rates were markedly higher compared with a single CA125, which produced a sensitivity of 56.1% at 64.15% specificity (72). Another study demonstrated notable detection sensitivities and specificities using a 10-gene panel in plasma (73).

The role of DNA methylation biomarkers in ovarian cancer is promising. However, progression towards clinical practice is hampered by the lack of detection techniques combining high accuracy with low cost. The main obstacles that are to be overcome are the standardization of analysis techniques and establishment of reliable reference values.

## 4. Treatment

### Chemoresistance

The current chemotherapy strategy in treating ovarian cancer patients involves a combination of a platinum- and a taxane-based therapy. While most ovarian cancer patients respond completely to chemotherapy, the majority of the initial responders eventually develop chemoresistance (74). In addition to mutations, DNA methylation-induced silencing of various drug response genes and pathways also facilitates the development of ovarian tumor cell drug resistance (75). It was shown that the silencing of *SFRP5,* which is a Wnt antagonist, by DNA hypermethylation was associated with platinum resistance of ovarian cancer (76). Similarly, hypermethylation of several genes such as *hMLH1*, the arginine biosynthesis-related gene *ASS1*, and *ESR2* (encoding the ER-b) are involved in platinum resistance (77–79). Platinum resistance has also been correlated with stage-progressive hypermethylation of the Methylation Controlled DNAJ (*MCJ*) gene which resulted in loss of gene expression and correlated with a poor response to chemotherapy (67). DAPK, which is a gene involved in apoptosis, has also been shown to be silenced in drug-resistant cancer due to methylation (80).

In addition to the loss of expression due to DNA methylation, it was shown that hypomethylation along with an increase in expression of the myelin and lymphocyte protein (*MAL*) gene is associated with platinum resistance (62). Hypomethylation and upregulation of the *ABCG2* multidrug transporter gene was also shown to occur during chemoresistance in two ovarian carcinoma cell lines (81). Based on the association of DNA methylation of specific genes with platinum sensitivity, it was shown that the hypomethylation-mediated activation of the cell growth-promoting pathways, PI3K/Akt, TGF-β and cell cycle progression, may contribute to cisplatin resistance in ovarian cancer cells (82).

At present, only two biomarkers of protein origin (CA125 and HE4) are considered as indicators of response to chemotherapy. Epigenetic markers may supplement these proteins possibly by increasing their sensitivity and specificity. DNA methylation biomarkers in particular, have several advantages over other biomarkers such as proteins, gene expression and DNA mutations, since they are stable, can easily be distinguished, and can be detected in specific DNA regions (CpG islands) (83). In the future, the overall DNA methylation profile of the resected ovarian tumor may may be used for the development of individually tailored treatment regimens (84).

### Epigenetic therapy

Unlike cancer-associated gene mutations, DNA methylation and other epigenetic modifications are potentially reversible. This makes epigenetic agents attractive candidates for disease prevention and resensitization to chemotherapeutic agents. Demethylation of tumor suppressor genes may have a positive effect in cancer progression, whereas the decrease of methylation of oncogenes which reactivate these genes, may have an adverse effect. There are two types of DNA methylation inhibitors: nucleoside and non-nucleoside analogues. Nucleoside analogues inhibit methylation when they are integrated into DNA and block the release of DNMTs by forming a covalent complex with these enzymes (85). They have been found to have clinical activities especially on hematopoietic malignancies (86–88). These inhibitors have been used to induce the re-expression of silenced TSGs caused by hypermethylation. Although aberrant promoter methylation is corrected by DNA methylation inhibitors, when the drug is stopped, the aberrant methylation and gene silencing is re-established (16). Non-nucleoside analogues are thus small molecular inhibitors that bind to the catalytic region of DNMTs and suppress translation.

Azacytidine and decitabine are the first two DNMT inhibitors approved for the therapy of myelodysplastic syndromes (13,31). Decitabine, a potent methylation inhibitor, has been shown to cause demethylation in numerous ovarian cell lines, reversing the silencing of several TSGs (89,90). Decitabine has also been reported to decrease cisplatin resistance in both ovarian cancer cells and a mouse xenograft through demethylation of the *hMLH1* promoter (91). Two clinical trials have provided evidence that azacytidine and decitabine are capable of reversing platinum resistance in ovarian cancer patients (92,93). However, DNMT inhibitors may simultaneously cause widespread genomic hypomethylation that potentially leads to genomic instability (94).

Histone deacetylation is a well-known epigenetic mechanism that also contributes to silencing of TSGs in cancer. While HDACIs and DNMTIs have demonstrated clinical activity as single-agent therapies for hematopoietic malignancies, DNA methylation and histone deacetylation often co-ordinately inhibit gene transcription, and restoration of the two silencing mechanisms may be necessary for maximal gene derepression (13). Treatment with a DNMTI/HDACI combination, in ovarian cancer cases, was synergistic for upregulation of the pro-apoptotic gene *TMS1*/*ASC*, in contrast to either agent alone (95). An earlier integrated microarray analysis demonstrated that a DNMTI/HDACI-combined treatment of ovarian cancer cells affects more genes that either agent individually (96). Conventional chemotherapy together with methylation inhibitors have also been examined in phase I/II clinical trials. Decitabine in combination with carboplatin demonstrated no significant improvement over platinum alone in an ovarian cancer study (97). Another similar study that uses low-dose decitabine plus carboplatin resulted in more disease responses and established *in vivo* biological activity in blood and tumor specimens of ovarian cancer patients (93). Carboplatin when combined with 5-azacytidine also showed encouraging results (92).

## 5. Conclusion

Epigenetic alterations such as DNA methylation are clearly involved in ovarian cancer initiation and progression. Global DNA hypomethylation and localized hypermethylation of specific gene promoters contribute to genome instability and transcriptional silencing of tumor suppressor genes, respectively. Early studies focused on the methylation patterns of single genes associated with tumorigenesis. However, newer genome-wide methods have identified a group of genes whose regulation is altered by DNA methylation during ovarian cancer progression. The profiling of DNA methylomes may provide new insight into the development of biomarkers with clinical value for cancer risk assessment, early detection, prevention and prognosis. Therapeutic agents that target methylation are already being tested for future use and have proven beneficial in other types of malignancies. This is an exciting and rapidly evolving area of research in which investigations may lead to the possible detection of interindividual drug response differences and their reversal.
